# Molecular Dynamics Simulations of Deformation Mechanisms in the Mechanical Response of Nanoporous Gold

**DOI:** 10.3390/ma13092071

**Published:** 2020-04-30

**Authors:** Mohammad Nasr Esfahani, Masoud Jabbari

**Affiliations:** 1 Department of Electronic Engineering, Faculty of Sciences, University of York, York YO10 5DD, UK; mohammad.nasresfahani@york.ac.uk; 2 Department of Mechanical, Aerospace and Civil Engineering, School of Engineering, University of Manchester, Manchester M13 9PL, UK

**Keywords:** nanopore gold, mechanical properties, pore shape, stress, molecular dynamics

## Abstract

The mechanical behaviour of nanoporous gold has so far been the subject of studies for bicontinuous morphologies, while the load transfer between ligaments is the primary challenge for using nanoporous structures—especially membranes with nanopores—in single-molecule sensors. This work studies the pore shape effect on deformation mechanisms of nanoporous gold membranes through molecular dynamics simulations. Tension and compression tests are carried out for nanoporous gold with circular, elliptical, square and hexagonal pore shapes. A significant pore shape effect on the mechanical properties is observed with distinct load transfer capabilities. A uniform stress transfer between ligaments constitutes a distinguished set of mechanical responses for structures with the hexagonal pore shape under tension, while a unique stress distribution in nanoporous with the circular pore shape introduces a high strength and ductile structure under compression. Further to shed light on the existing experimental observations, this work provides a comprehensive study on load transfer capabilities in the mechanical behaviour of nanoporous gold for sensing applications.

## 1. Introduction

The extreme surface-to-volume ratios in nanoporous (np) structures lead to unique adoption and interaction properties for sensing [1], bioengineering [2,3] and fuel cell [4] technologies. Among those aspects explored for np materials, bioengineering has been highlighted with a considerable focus on bio-molecular detections [2,5]. The fundamental mechanism to utilize np structures for cell signalling, selective ion channels, RNA translation and protein screening is based on the monitoring ion currents and forces during the interaction of molecules and pores. Force detections in np structures enable high-resolution bio-sensing. For example, the recognition of DNA about 28 aM through np materials is demonstrated through using electrostatic interactions [6]. The binding between biomolecules and np ligaments is another example of the selective detection of bioanalytes at ultra-low concentrations [7]. Therefore, understanding the mechanical properties of np structures is the immediate challenge to study the translocation process in bio-molecular detections [8].

The structure of np materials can be considered as a network of nanowires. A wide range of studies has examined the mechanical properties of nanowires [9] as well as np materials [10,11,12,13,14,15] exhibiting a considerable size-dependent mechanical behaviour. In all the aforementioned studies, the surface energy is determined to be the main reason of the size effect in np structures; this has drawn attention to the modification of the scaling law. While pore size and relative density are found to be effective parameters to change the scaling law, recent studies demonstrate the influence of pore shape on the mechanical properties of np silicon [16,17]. Moreover, network connectivity has shown to have important influence on the scaling law of np metal foams [18]. Among porous metals, np gold (Au) has introduced unique features in stability, conductivity and biocompatibility for sensing and actuation purposes with significant size-dependent mechanical properties [19,20].

A wide range of experimental techniques has been developed so far to study the mechanical behaviour of np Au focusing on ligament size and relative density [21,22,23]. Increasing the elastic modulus [24] and yield strength [25] is reported as a result of ligament diameter size reduction. This size dependency is linked to various factors including surface effects, density increase and surface defects. Although a yield strength of about 1.5 GPa for an individual ligament in np Au is measured to represent a shear strength about the theoretical limit [26,27], the lower strength and elastic modulus measured in some studies compared to the theoretical level are attributed to the network connectivity of np Au [28]. Similar contrast of the size-dependent fracture strength of np Au [29,30] is linked to the network structure rather than the microscopic brittleness [31,32]. Those contrasts between experimental measurements and theoretical approaches regarding the mechanical strength [26,27,28,29,30] and deformation behaviour [14] have become the motivation of computational techniques to study effective parameters on the mechanical properties of np Au. The theory of surface elasticity [33], a unit–cell micromechanical model [13] and finite element methods [34] have been developed to implement the surface effect on the mechanical behaviour of np structures. Molecular dynamics (MD) simulations, on the other hand, are considered as a powerful approach to study the mechanical properties of np Au [11,35,36,37,38,39,40,41]. While plastic deformation and defect formation are the main focus of atomic simulations in np structures [37,38,39,41], the surface effect on the asymmetric mechanical behaviour is studied in order to modify the scaling law for np Au [11,40].

The ligament size and pore density have so far been the focus of studies to understand the mechanical behaviour of np Au with bicontinuous pore shapes [11,35,37,38,39,40,41,42], while a recent study demonstrated a significant pore shape effect on the mechanical behaviour of np silicon [17]. Moreover, the force transfer mechanism between ligaments is a fundamental aspect of np Au in general, especially for sensing applications [8,19]. It is, therefore, the aim of this work to use MD simulations for investigating the pore shape effect on the load transfer mechanisms and deformation behaviour of np Au membranes. In the remainder of this work, the simulation method will be described first. Then, tensile and compressive properties will be studied for circular, elliptical, square and hexagonal pore shapes. The mechanical behaviour will be analysed through studying the stress distribution and deformation mechanisms of np Au. This work is concluded by a discussion on further insight of np Au for reliable sensing applications.

## 2. Simulation Methods

**Figure 1:**
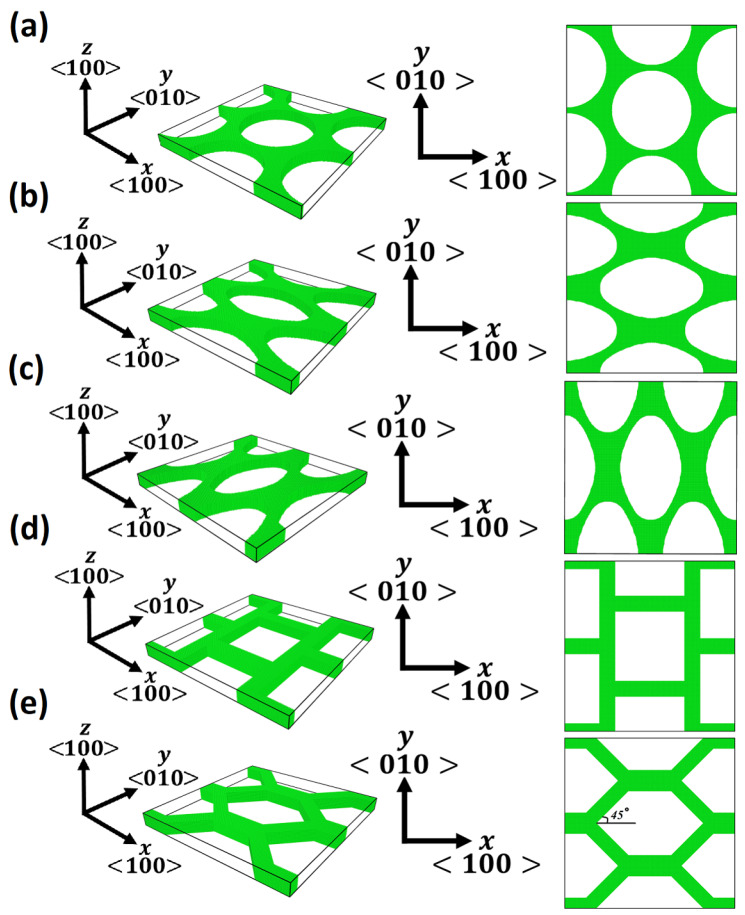
Snapshot of simulation unit–cells representing np structures with (**a**) circular, (**b**) H-ellipse, (**c**) V-ellipse, (**d**) square and (**e**) hexagonal pore shapes have a relative density $\bar{\rho }=0.343$ and an average ligament size of 5 nm.

In this study, the influence of pore shape on deformation mechanisms of np Au membranes (2-dimensional structures) is studied through MD simulations. The Sandia-developed simulation code (LAMMPS) is used to study the mechanical behaviour of np Au [43]. The embedded atomic method (EAM) potential, Φ, is considered to model gold single crystal in a form of traction between embedded energy, $F_{m}$, and electrostatic energy, $\phi_{mn}$, of cores as

(1) $$\Phi =\sum_{m=1}^{N}F_{m}\left(\sum_{n\neq m}^{N}\rho_{n}\left(r_{mn}\right)\right)+\frac{1}{2}\sum_{n\neq m}^{N}\phi_{mn}\left(r_{mn}\right)$$

where $r_{mn}$ is the distance between atoms *m* and *n*, $\rho_{n}$ is the contribution of atom *n* into the electron density of atom *m*, and *N* is the total number of bonds for a fully coordinated atom in the fcc crystal. A unit cell of 52 nm × 52 nm × 4 nm along <100> crystal direction is considered with periodic boundary conditions (Figure 1). The porous media is modelled through considering different pore shapes, including circle, horizontal ellipse (H-ellipse), vertical ellipse (V-ellipse), square and hexagon with a same relative density of 0.343 and an average ligament size of 5 nm. These geometrical parameters are selected based on an average design studied previously through a series of computational [11,35] and experimental [10,27,29] works. The relative density, $\bar{\rho }=\frac{\rho_{p}}{\rho_{B}}$, stands for the ratio of the density of the porous structure, $\rho_{p}$, to the density of the bulk material, $\rho_{B}$. The major axis of the ellipse aligned and orthogonal to the loading direction (*x*-direction) represents H-ellipse and V-ellipse pore shapes, respectively. First, the energy of each simulation is minimised through the conjugate gradient method with a time step of 1 fs. After a thermal relaxation at a temperature of 10 *K* for 50 ps, uniaxial deformation (tension or compression) is carried out in *x*-direction at a strain rate of $10^{-4}\;ps^{-1}$ in a Nose-Hoover [44] isothermal-isobaric (NPT) ensemble at a temperature of 10 *K* and a pressure of 0 bar. The low temperature is selected to eliminate thermally activated processes during the mechanical deformation. Parameters of this model are validated through replicating the work presented previously for np Au [37].

Stress analysis is carried out through calculating the von Mises stress, $S_{v}$, as

(2) $$S_{v}=\sqrt{\frac{3}{2}s_{ij}s_{ij}}$$

(3) $$s_{ij}=\pi_{ij}-\pi_{kk}\delta_{ij}/3$$

where the atomic stress tensor, $\pi_{ij}$, is calculated for a quasistatic system without any thermal oscillations as [45]

(4) $$\pi_{ij}=\frac{1}{2\Omega_{0}}(\sum_{m=1}^{N}\sum_{n\neq m}^{N}\frac{1}{r_{mn}}\frac{\partial \Phi (r_{mn})}{\partial r}\left(v_{mn}^{i}v_{mn}^{j}\right))$$

in which $\Omega_{0}$ is the atomic volume in an undeformed atomic system, $v_{mn}^{j}=v_{m}^{j}-v_{n}^{j}$, and $v_{m}^{j}$ is the position of atom *m* along direction *j*. Further to the stress analysis, deformation mechanism is studied through calculating the centro-symmetry parameter (CSP) as

(5) $$c_{i}=\sum_{\alpha =1}^{N/2}|r_{\alpha }+r_{\alpha +N/2}{|}^{2}$$

where $r_{\alpha }$ and $r_{\alpha +6}$ are the vectors of the six nearest neighbour atoms in the fcc crystal.

## 3. Results and Discussion

### 3.1. Tensile Test

**Figure 2:**
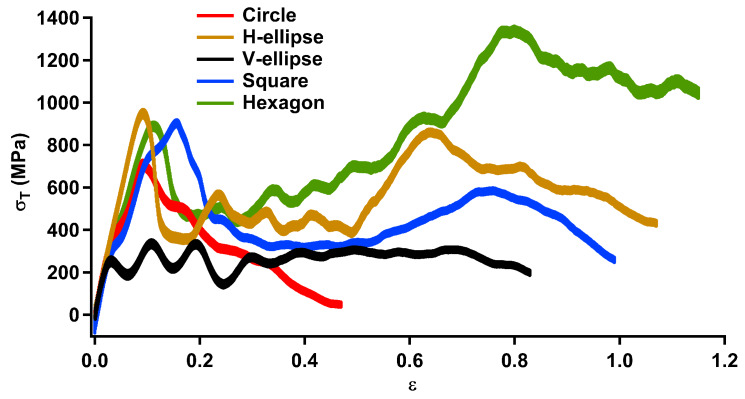
Tensile stress–strain graph for np Au with pore shapes of circle, H-ellipse, V-ellipse, square and hexagon. All np structures have the relative density of $\bar{\rho }=0.343$ and an average ligament size of 5 nm.

In the first stage, the engineering strain, ε, is calculated as the ratio of the change in the simulation length along the deformation direction (*x*-direction) over the initial unit–cell length after relaxation. Then, the normal tensile stress, $\sigma_{T}=\sigma_{11}$, is obtained through calculating the pressure at the unit–cell boundary in *x*-direction. Figure 2 shows the stress–strain curves for various pore shapes with $\bar{\rho }=0.343$ and an average ligament size of 5 nm. Both $\bar{\rho }$ and the ligament size are based on the already published data from computational [11,35] and experimental [10,27,29] studies. The change in stress is linear until the tensile yield stress, $\sigma_{Y}^{T}$, while the stress changes non-linearly afterwards to the ultimate tensile stress, $\sigma_{U}^{T}$. After the initiation of ligament necking, the stress decreases to the complete ligaments break. Figure 2 exhibits a significant pore shape dependence in the mechanical behaviour of np Au. The stress–strain curves are used to measure the mechanical properties of np structures that is summarised in Table 1. The linear slope of the stress over the strain for ε<0.5% is utilized to calculate the tensile elastic modulus, $E^{T}$. The initiation of non-linear stress–strain curve is considered as the point to compute $\sigma_{Y}^{T}$ and the tensile yield strain, $\varepsilon_{Y}^{T}$. The ultimate tensile strain, $\varepsilon_{U}^{T}$, and $\sigma_{U}^{T}$ are calculated at the maximum point after yielding. The tensile fracture strain, $\varepsilon_{f}^{T}$, is measured at the complete fracture of a ligament in the unit–cell. Then, the tensile fracture toughness is computed through numerical integration of the stress–strain curve, $\Pi^{T}=\int_{0}^{\varepsilon_{f}^{T}}\sigma \left(\varepsilon \right)d\varepsilon$.

An elastic modulus of about 9.9–12.5 GPa is achieved by changing the pore shape. In contrast to the elastic modulus, a significant pore shape effect is obtained for the yield strength and the ultimate strength. The utmost $\sigma_{Y}^{T}$ and $\varepsilon_{Y}^{T}$ are obtained for the H-ellipse pore shape about 510 MPa and 4.1%, respectively, while the minimum yield strength and yield strain are observed for the V-ellipse pore shape about 180 MPa and 1.7%, respectively. Similar to the yield strength, the H-ellipse pore shape has a maximum $\sigma_{U}^{T}$ of 968 MPa. The lowest $\sigma_{U}^{T}$ and $\varepsilon_{U}^{T}$ are observed for the V-ellipse pore shape of about 273 MPa and 3.1%, respectively. A similar considerable pore shape effect is observed for the fracture behaviour of np Au. The hexagonal pore shape constitutes the utmost level of $\varepsilon_{f}^{T}$ and $\Pi^{T}$ about 115% and 0.806 $J/mm^{3}$, respectively, while the circular pore shape has the lowest fracture strain of 47% with a toughness of 0.148 $J/mm^{3}$. The obtained elastic modulus is higher than those reported in tensile measurements with E<4 GPa for np Au with $\bar{\rho }\approx 0.35$ [14,30], which can be linked to the higher ligament size in the experimental studies with one-order-of-magnitude scale difference. This can be recognised by a comparison to the elastic modulus about 7–11 GPa obtained in compression [27] and nanoindentation [23,26,32] tests with the same ligament size. In addition to experimental observations, the obtained tensile behaviour can be compared with previous computational studies as well. The tensile elastic modulus is comparable with np Au with bicontinuous morphologies representing a negligible pore shape effect in the elastic behaviour [11]. Similar to the tensile elastic modulus, $\sigma_{Y}^{T}$ is about those reported for bicontinuous structures with a tensile yield strength between 200 and 250 MPa. [11,39].

**Figure 3:**
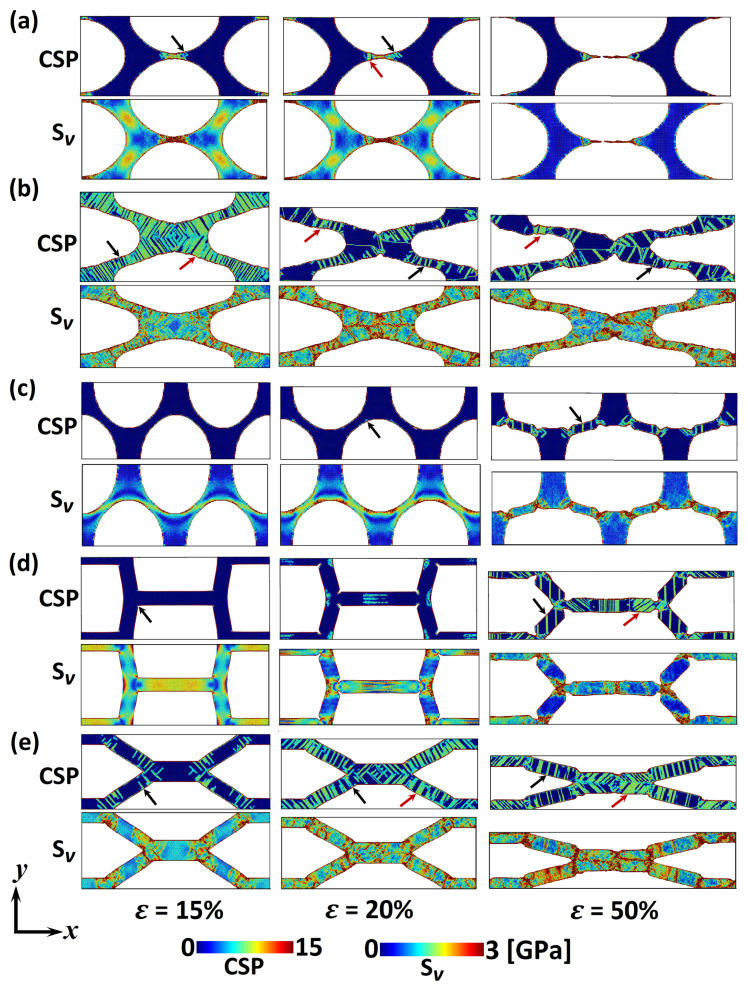
CSP and von Mises stress, $S_{v}$, for np Au with (**a**) circular, (**b**) H-ellipse, (**c**) V-ellipse, (**d**) square, and (**e**) hexagonal pore shapes deformed in *x*-direction with tensile strain of 15%, 20% and 50%. Black and red arrows indicate dislocation (DL) and stacking fault (SF) defects, respectively. Half of the simulation box along the *y*-direction is represented here due to the symmetrical deformation.

After comparing the mechanical properties with previous studies, the stress distribution and deformation behaviour under tension will be investigated for each pore shape to provide more insight into load transfer capabilities of np Au. The von Mises stress, $S_{v}$, distribution (Equation (2)) and CSP (Equation (5)) are calculated for an averaged rang of the tensile strain of 15%, 20% and 50%. Figure 3 demonstrates CSP and von Mises stress distribution for np Au with various pore shapes deformed in *x*-direction. In this figure, half of the simulation unit cells (Figure 1) in *y*-direction is exhibited due to the symmetrical deformation. Dislocation (DL) and stacking faults (SF) defects are indicated through black and red arrows, respectively. Figure 3 confirms that there is a considerable pore shape effect in the tensile deformation behaviour of np Au. Starting from ε=15%, stress concentration is observed at ligaments in *x*-direction and ligaments junctions, respectively, in the circular and square pore shapes leading to a local deformation (Figure 3a,d). In contrast, the stress concentrates along diagonal ligaments of the V-ellipse pore shape without any noticeable defect formation (Figure 3c). While the circular, square and V-ellipse pore shapes exhibit stress concentration, the von Mises stress distribution replicates shear stresses across ligaments of the H-ellipse and hexagonal pore shapes. This stress profile creates considerable DL and SF defects in the structure (Figure 3b,e). Defect density is the main difference in the plastic deformation of those pore shapes. This contrast among the deformation behaviour of np Au with different pore shapes is enhanced through increasing the tensile strain. Further, strain energy to strain levels of 20% and 50% imposes a local deformation at stress concentration regions in np Au with circular, square and V-ellipse pore shapes, while a uniform plastic deformation along ligaments is observed in the H-ellipse and hexagonal pore shapes.

After comparing the deformation behaviour of various pore shapes, the tensile properties presented in Figure 2 can be analysed based on the load transfer mechanism (Figure 3). For example, the higher $\sigma_{Y}^{T}$ and $\sigma_{U}^{T}$ in the H-ellipse pore shape can be linked to the uniform stress distribution compared to the other pore shapes, while the stress concentration on the pore surface leads to the lower tensile strength of the V-ellipse pore shape. Similar discussion can be made for the ultimate tensile strain as well. The higher $\varepsilon_{U}^{T}$ in the square pore shape can be traced back to the uniaxial tension in ligaments compared to the shear deformation in the H-ellipse and hexagonal pore shapes. Beside the tensile strength, the unique fracture toughness in the hexagonal pore shape can be linked to its stress transfer capabilities, where the shear stress is distributed along ligaments with less DL activation compared to the H-ellipse pore shape. Moreover, the shear stress transition between ligaments in np with hexagonal pore shape prevents the formation of stress concentrations observed in the square and V-ellipse pore shapes.

The deformation behaviour shown in Figure 3 can be compared with experimental observations. For example, here, MD simulations exhibit a local plastic deformation at ligament junctions mainly in the V-ellipse pore shape (Figure 3c), similar to experimental observations at nodes of bicontinuous np Au under tension [10]. This raises the attention to consider the V-ellipse profile as a dominant pore shape in np Au with random bicontinuous structures. Furthermore, Figure 3 demonstrates the dominance of DL and SF defects on the tensile deformation of np Au. Similar mechanisms on the plastic behaviour of Au nanowires—as ligaments in np structures—are reported under tensile tests [46,47]. Therefore, the high DL density of the H-ellipse pore shape (Figure 3b) can be linked to the stress concentration at surface steps. The stress concentration at surface steps induces preferential regions for dislocation nucleation [46].

### 3.2. Compressive Test

**Figure 4:**
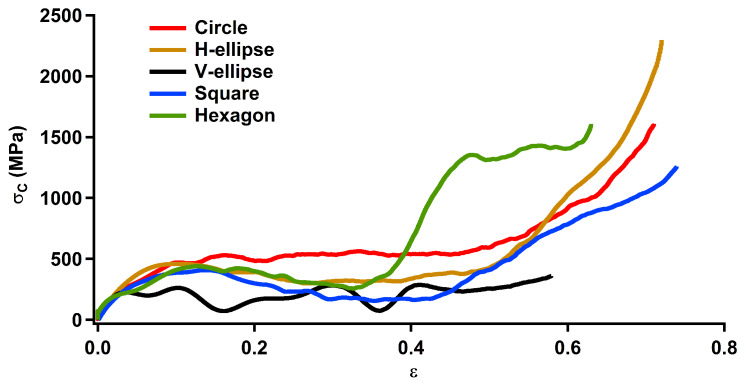
Compressive stress–strain graph for np Au with pore shapes of circle, H-ellipse, V-ellipse, square and hexagon. All np structures have the relative density of $\bar{\rho }=0.343$ and an average ligament size of 5 nm.

After the tensile test, the pore shape effect on the compressive behaviour of np Au is studied with the same approach. In the first step, stress–strain curves are obtained for the compressive test of np structures (Figure 4). The change in stress is linear until the compressive yield point, $\sigma_{Y}^{C}$, where the stress changes non-linearly to the ultimate compressive stress, $\sigma_{U}^{C}$. Then, the stress decreases to a minimum point, where densification starts with increasing the stress. Figure 4, once again, demonstrates the significance of the pore shape effect in the mechanical behaviour of np Au. The compressive properties of np structures are listed in Table 2. The compressive elastic modulus, $E^{C}$, is calculated as the linear slope of the stress over strain for ε<0.5%. The compressive yield strain, $\varepsilon_{Y}^{C}$, and $\sigma_{Y}^{C}$ are measured at the point of linear to nonlinear stress–strain curves. The ultimate compressive strain, $\varepsilon_{U}^{C}$, and $\sigma_{U}^{C}$ are calculated at the maximum stress after the yield point. The compressive densification strain, $\varepsilon_{D}^{C}$, is estimated at the initiation of densification. The compressive fracture strain, $\varepsilon_{f}^{C}$, is estimated at the void collapse. Then, the compressive fracture toughness is computed through numerical integration of the stress–strain curve, $\Pi^{C}=\int_{0}^{\varepsilon_{f}^{C}}\sigma \left(\varepsilon \right)d\varepsilon$.

The compressive mechanical behaviour exhibits an elastic modulus of about 10 GPa with a negligible pore shape effect, which is comparable with the tensile elastic modulus. A considerable pore shape dependency on the compressive yield strength is demonstrated with the utmost and the minimum of 160 MPa and 95 MPa for the circular and square pore shapes, respectively. Similarly, the ultimate $\varepsilon_{Y}^{C}$ of 1.6% is obtained for the circular pore shape, while the square pore shape constitutes the lowest yield strain of 1.0%. The V-ellipse pore shape has the lowest $\sigma_{U}^{C}$ and $\varepsilon_{U}^{C}$ of 222 MPa and 3.4%, respectively. The maximum $\sigma_{U}^{C}$ of 471 MPa and $\varepsilon_{U}^{C}$ of 14% are obtained for the circular and hexagonal pore shapes, respectively. After a uniform plastic deformation under compression, densification initiates through reducing the relative density and void collapse. The utmost $\varepsilon_{f}^{C}$ of 74% and $\Pi^{C}$ of 0.432 $J/mm^{3}$ are obtained for the hexagonal and circular pore shapes, respectively, while the V-ellipse pore shape exhibits the minimum $\varepsilon_{f}^{C}$ of 58% and $\Pi^{C}$ of 0.12 $J/mm^{3}$.

Similar to the tensile properties, $E^{C}$ is comparable with experimental measurements [23,26,27,32] and computational studies [11,37] of np Au with bicontinuous pore shapes reporting a compressive elastic modulus between 7 and 11 GPa. This demonstrates a negligible pore shape effect on the elastic modulus of np structures. A compressive yield strength of 75 MPa to 150 MPa is predicted for various pore shapes (Figure 4), which is on the same level of those obtained by experimental measurements reporting a yield strength about 100–145 MPa for np Au with bicontinuous pore shapes with a ligament size of 10 nm and a relative density of 0.35 [22,23,27,32]. Therefore, this variation of the yield strength in experimental observations can be linked to the pore shape effect in np structures with bicontinuous morphologies as a combination of circular and elliptical pore shapes [28]. Figure 4 can be compared with previous computational studies as well. For example, atomic simulations estimate a yield strength about 100 MPa to 150 MPa for np Au with bicontinuous pore shapes [11], which is comparable with structures having circular and elliptical pore shapes with $\sigma_{Y}^{C}\approx$ 150 MPa.

Although a similar elastic modulus is predicted under tension and compression, the tensile yield strength is about 1.3 times higher than the compressive yield strength for the V-ellipse pore shape. The tension-compression ratio for the yield strength is about 3.4 for the H-ellipse pore shape. This asymmetry on the mechanical behaviour is observed for the ultimate strength as well. The lowest tension-compression ratio of 1.2 for the ultimate strength is obtained for the V-ellipse pore shape, while the hexagonal pore shape constitutes the utmost tension-compression ratio of 2.2 for the ultimate strength. Although the tensile yield strength and the tensile ultimate strength are higher than those obtained under the compression test, the compressive fracture toughness of the circular pore shape is about seven times higher than the tensile fracture toughness, in contrast to the other pore shapes. Comparing the mechanical behaviour of np Au listed in Table 1 and Table 2 demonstrates a tension-compression asymmetry for various pore shapes. While this asymmetry was predicted previously for np structures with bicontinuous pore shape due to the surface stress [11,40], here, the pore shape effect on the tension-compression asymmetry is demonstrated, for the first time, for np Au.

**Figure 5:**
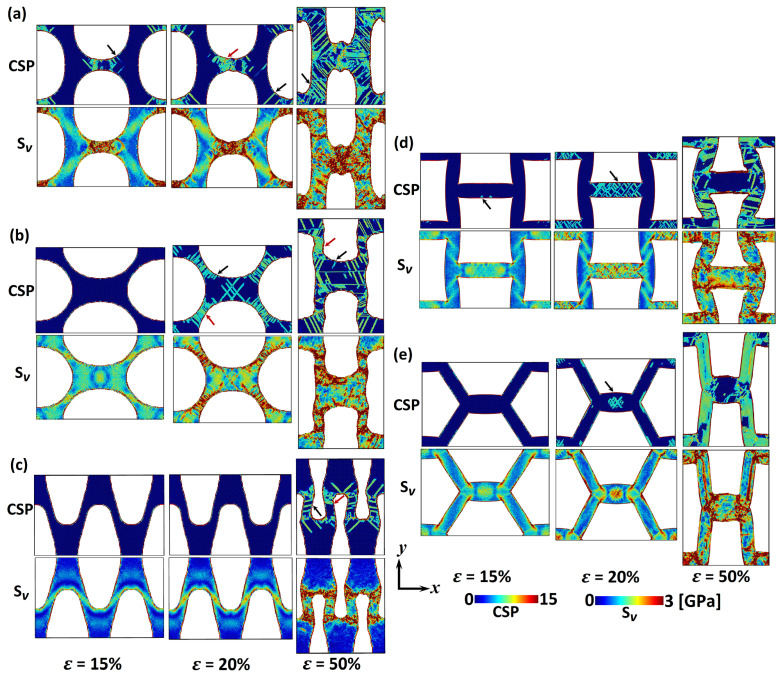
CSP and von Mises stress, $S_{v}$, for np Au with (**a**) circular, (**b**) H-ellipse, (**c**) V-ellipse, (**d**) square, and (**e**) hexagonal pore shapes deformed in *x*-direction with compressive strain of 15%, 20% and 50%. Black and red arrows indicate dislocation (DL) and stacking fault (SF) defects, respectively. Half of the simulation box along *y*-direction is represented here due to the symmetrical deformation.

Similar to the tensile study presented in the previous section, the stress distribution and deformation behaviour are studied through calculating the von Mises stress (Equation (2)) and CSP (Equation (5)), respectively. Figure 5 demonstrates CSP and von Mises stress distribution for np Au with various pore shapes compressed with strain levels of 15%, 20% and 50%. Results exhibit a significant pore shape effect in the compressive deformation behaviour of np Au. Let us start with ε=15%, where the strain energy induces a considerable plastic deformation in the circular pore shape without any noticeable defects in the other pore shapes. This can be linked to the stress concentration across ligaments in *x*-direction for the circular pore shape. Although the von Mises stress distribution in the circular, V-ellipse and square pore shapes is similar to the tensile tests (Figure 3), the hexagonal pore shape exhibits a stress concentration at ligaments junctions in contrast to the shear stress profile observed under the tensile strain (Figure 3e). Increasing the strain level to 20% constitutes a deformation with insignificant defect nucleation in the V-ellipse pore shape and defects growth in the other pore shapes. In this strain level, defects are mainly distributed across diagonal ligaments in the H-ellipse pore shape in contrast to ligaments in *x*-direction for the circular, square and hexagonal pore shapes. This deformation mechanism changes dramatically at the compressive strain of 50%. A stress concentration at ligaments junctions is observed with a significant defect formation in the V-ellipse pore shape. The other change is defects growth in diagonal ligaments of the circular, square and hexagonal pore shapes at ε=50%. However, the defect formation in compressed np structures (Figure 5) is found to be different than those observed in the tensile tests (Figure 3), deformation mechanisms are mainly controlled through DL and SF defects regardless of the pore shape and loading method. The main plastic deformation system in the Au bulk crystal is twins with the nucleation and propagation of <112> DL on {111} planes, while the surface contribution in nanoscale structures changes this deformation mechanism into distributed SF defects [9,46,47]. Therefore, the high surface area in np structures leads to a plastic deformation mechanism mainly through DL and SF defects, while Figure 3 and Figure 5 exhibit the influence of the pore shape on defects nucleation and growth through stress distribution between ligaments.

After studying the pore shape effect on the deformation behaviour, the mechanical properties presented in Figure 4 can be analysed based on the stress transfer mechanisms (Figure 5). For example, the lower $\sigma_{Y}^{C}$ at the square pore shape can be linked to the stress concentration at pore corners. Furthermore, the higher $\sigma_{U}^{C}$ in the circular pore shape can be traced back to the uniaxial compressive stress in ligaments compared to the stress concentration in the other pore shapes. A similar discussion can be raised regarding the stress concentration at nodes of the V-ellipse pore shape leading to the lower fracture toughness. In addition to the mechanical behaviour analysis, Figure 5 can be compared with experimental observations as well. For example, a local deformation is observed at ligaments junctions of the V-ellipse pore shape (Figure 5c) similar to bicontinuous morphologies reported previously [10]. The shear stress and defects formation are mainly along <110> crystal orientation (Figure 5) comparable with experimental observations in np Au with bicontinuous morphologies as well [10,31].

Although various attempts have been carried out to understand the mechanical behaviour of np Au for bio-molecular detection and energy conversion applications, bicontinuous morphologies as a combination of circular and elliptical pore shapes have been studies so far focusing on the relative density and pore size [10,11,21,23,24,25,26,27,30,31,35,37,40,41]. This work demonstrates, for the first time, the pore shape effect in the mechanical behaviour of np Au through determining the stress transfer mechanism between ligaments. A rigorous stress transfer in np Au can be utilized to monitor bio-molecular translocation processes [8]. This necessitates further studies through fabricating various morphologies— especially two-dimensional np structures— to control molecular transferring processes [2,3]. Beside a comprehensive determination of the pore shape effect on the deformation behaviour of np Au, there are few limits associated with the presented work to be considered for further investigations. For example, design parameters studied here are for np Au with $\bar{\rho }=0.343$ and an average ligament size of 5 nm along <100> crystal orientation, while size and crystal orientation control the mechanical behaviour [11,39]. Moreover, the role of thermal processes on deformation mechanisms remains to be addressed. Although the scaling law has been modified based on ligament size and relative density for np structures, recently the influence of hexagon angle on the mechanical behaviour of honeycomb np silicon is studied for further modification of the scaling law [17]. Therefore, the pore shape effect needs to be considered on the scaling law formulation. The size dependence mechanical behaviour of np Au is modelled through theoretical studies [33,34], while multiscale modelling approaches can be developed to implement the surface energy into the mechanical analysis at finite temperature [48].

## 4. Conclusions

This work addresses the pore shape effect on stress transfer and deformation mechanisms of np Au membrane through utilizing MD simulations. The mechanical behaviour of gold np structures under tension and compression is studied for circular, elliptical, square and hexagonal pore shapes with a relative density of 0.343 and an average ligament size of 5 nm. In the first stage, stress–strain graphs are utilized to determine the mechanical properties including the elastic modulus, the yield strength, the ultimate strength and the fracture toughness. Then, deformation mechanisms in the mechanical response are studied through a comprehensive analysis of the von Mises stress and centro-symmetry parameter. The main outcomes can be summarised as follows:

- The pore shape effect is negligible for the elastic modulus, while the influence of the pore shape is significant on the mechanical strength and fracture behaviour of np Au.
- A uniform stress distribution along ligaments leads to a higher tensile strength as well as fracture toughness in the H-ellipse pore shape compared to the other pore shapes.
- A higher compressive strength is observed in the circular pore shape with uniformly distributed stress in ligaments compared to the other pore shapes with concentrated stresses.
- The pore shape effect in asymmetric tension-compression is observed for np Au. The utmost tension-compression ratio of 3.4 is obtained for the yield strength of the H-ellipse pore shape, while the V-ellipse pore shape has the lowest tension-compression ratio of 1.3. The tensile strength is found to be higher than the compressive strength for all pore shapes, while the compressive fracture toughness is seven times higher than the tensile fracture toughness in np Au with pore shape of circle.

Although relative density and ligament size for nanoporous Au have been studied so far for bicontiuous pore shapes, this work provides a comprehensive analysis on the role played by the pore shape on load transfer mechanisms in the mechanical response of np Au. The significant pore shape effect on the mechanical behaviour of np structures raises the need to modify the scaling law [17].

## Figures and Tables

**Table 1:** Summarised uniaxial tensile properties of np Au with pore shapes of circle, H-ellipse, V-ellipse, square and hexagon.

Pore Shape	$E^{T}$ (GPa)	$\sigma_{Y}^{T}$ (MPa)	$\varepsilon_{Y}^{T}\%$	$\sigma_{U}^{T}$ (MPa)	$\varepsilon_{U}^{T}\%$	$\varepsilon_{f}^{T}\%$	$\Pi^{T}\left(J/{\mathrm{mm}}^{3}\right)$
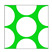	11.8	255	2.0	728	9.1	47	0.148
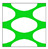	12.4	510	4.1	968	9.2	107	0.59
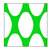	9.9	180	1.7	273	3.1	83	0.21
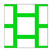	12.1	245	2.1	920	15.7	99	0.44
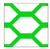	10.3	275	2.5	908	11.3	115	0.806

**Table 2:** Summarised uniaxial compressive properties of np Au with pore shapes of circle, H-ellipse, V-ellipse, square and hexagon.

Pore Shape	$E^{C}$ (GPa)	$\sigma_{Y}^{C}$ (MPa)	$\varepsilon_{Y}^{C}\%$	$\sigma_{U}^{C}$ (MPa)	$\varepsilon_{U}^{C}\%$	$\varepsilon_{D}^{C}\%$	$\varepsilon_{f}^{C}\%$	$\Pi^{C}\left(J/{\mathrm{mm}}^{3}\right)$
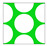	10.1	160	1.6	471	10.3	45.8	71	0.432
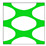	10.2	150	1.5	460	10.4	46.7	72	0.406
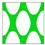	9.9	140	1.3	222	3.4	36.0	58	0.12
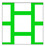	9.5	95	1.0	439	12.7	32.8	63	0.42
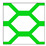	9.1	128	1.3	407	14	42.6	74	0.313
